# A Pseudo Tumor of the Abdomen

**Published:** 1880-01

**Authors:** Lunsford P. Yandell


### A PSEUDO TUMOR OF THE ABDOMEN.

By LUNSFORD P. YANDELL, M.D.

Mr. H., a powerfully-built, fleshy man, aged seventy, in comfortable circumstances, came to me early in June, in 1878, seeking relief from a dropsy of the legs, and general debility. His temperature was normal; his complexion pale and pasty; his face puffy; and he was short of breath. His pulse was rapid and feeble; his appetite variable; his digestion deranged, vomiting being frequent. Nocturnal micturition occurred often, and his sleep was broken by repeated calls to the urinal. The amount of water passed was stated to be normal, or but little augmented. Headache was of almost daily occurrence, and was frontal in its location. He had a slight but noticeable bronchitis. Hearing was defective, but this antedated the urinary symptoms. The vision was much impaired, and sometimes it was insufficient to enable him to read; sometimes the letters seemed doubled, sometimes blurred. The bowels were in the main constipated, and attended by pain in evacuation, but not infrequently diarrhœa or dysentery supervened. Mr. H. had always led a temperate, indeed, an abstemious life, and his liver evinced no signs of derangement. The spleen was normal. The heart was somewhat irregular in its action, losing a stroke every six or eight, or twenty or thirty beats, without uniformity, and there were present evidence and history of valvular insufficiency of long standing. There was, however, no ground for charging the dropsy to the disability of the heart. The patient had been "complaining" for six or eight years. Has had hemorrhoids forty years. More or less dysuria had existed three years. Three months before consulting me, coincident with a severe cold, swelling of the legs was observed. This steadily increased, giving much inconvenience by weight, pressure on the skin, and stiffness of the knees.

Suspecting albuminaria and discovering no special cause for the disease, the patient was put on bromide of potash, ergot, iron and milk diet, and elaterium was given to remove the serous effusion. Large watery discharges followed the elaterium, and the leg symptoms diminished satisfactorily; but in a few days decided mental abberration came on, in consequence, possibly, of the elaterium or the ergot. I have known it brought on by each of these medicines, and both were discontinued.

A careful analysis of the urine, to my surprise, failed to discover any albumen, sugar, or other abnormal constituent. The water was found limpid and excessive in quantity.

The patient was now stripped and submitted to a minute search for abnormities which might account for his troubles.

In the lower portion of the abdomen a round tumor as large as a three-year old child's head was discovered. It was immovable, solid, fibroid in feeling, and painless. The thick abdominal walls interfered greatly with the sense of touch. Mr. H. and his wife both declared the tumor had never before been suspected. It was the wife's custom to assist her husband in his bathing, and he had often complained of discomfort on pressure and rubbing of the abdomen during the process of ablution, but no swelling was perceived. At my request Dr. Coleman Rogers was called in consultation. On hearing the history of the case he, like myself, suspected albuminuria until the analysis of the urine was mentioned and the abdominal tumor was pointed out. The edema of the limbs was of course due to the pressure of the tumor, and the question to solve was the nature of this. The sex of the patient excluded uterine or ovarian origin, and the testicles were in their right place. Its physical characters precluded its being a fecal accumulation, a floating kidney, or an aneurism. There was no perceptible sign of fluid or gas.

A fibroid tumor attached to the bladder was now what we guessed. An examination of the bladder by means of a sound was determined on, but the excessive tenderness of the urethra and the patient's nervousness rendered the introduction of the sound impossible. A small Nelaton's catheter was with difficulty passed in, and resulted in the withdrawal of a few drops of blood and pus and about two gallons of clear urine. Subsequent digital examination detected considerable enlargement of the prostate gland and neck of the bladder.

The patient expressed himself as much relieved. He was put upon tonics and constructives; a fruit, meat, and milk diet was ordered, and for some weeks the urine was drawn off morning and evening, each time a gallon, more or less, coming away. During the third week a painful orchitis supervened, which ended in abscess. This being opened, a considerable quantity of healthy pus was evacuated, and the wound gradually healed. A small amount of matter after each catherterization came away, and this has continued. The instrument was intrusted to the patient after he was taught its use, and he has used it since three or four times in the twenty-four hours. The orchitis was probably consequent on the irritation of the catheter and his depraved health. The urine at present is less abundant than formerly, but is still abnormal in quantity. The patient's health was rapidly restored, and he is now as comfortable and vigorous as most men of his age. His only annoyance is in the use of the catheter.

The symptoms connected with the digestive apparatus, nervous system, etc., were due in all likelihood to the retention of the urine, its damming up, and resorption.

A case in most respects similar to this occurred in my practice nine years ago. At my suggestion Prof. Austin Flint was consulted. Death occurred within a week after Prof. Flint saw the patient. A post-mortem examination disclosed a saculated condition of the bladder. A stricture just inside the viscus had led to enormous distension and thickening of its walls. Its walls were nearly two inches in thickness and it possessed a capacity of more than two gallons.—*Louisville Medical News.*

